# Trend in Annual Risk of Tuberculous Infection in North India

**DOI:** 10.1371/journal.pone.0051854

**Published:** 2012-12-26

**Authors:** Kamal Chopra, Vineet K. Chadha, Jitendra Ramachandra, Nishi Aggarwal

**Affiliations:** 1 Director's Office, New Delhi Tuberculosis Center, New Delhi, India; 2 Epidemiology & Research Division, National Tuberculosis Institute, Bangalore, Karnataka, India; 3 HRD Division, National Tuberculosis Institute, Bangalore, Karnataka, India; 4 Statistics Department, New Delhi Tuberculosis Center, New Delhi, India; Royal Melbourne Hospital, Australia

## Abstract

**Setting:**

Six selected districts in Northern India.

**Objectives:**

To find out the trend in Annual risk of tuberculous infection (ARTI) in north India.

**Study Design:**

Two rounds of community level surveys were conducted during 2000–2001 and 2009–10 respectively. Representative samples of children 1–9 years of age were tuberculin tested and maximum transverse diameter of induration was recorded in mm at about 72 hours. ARTI was computed from the estimated Prevalence of infection using mirror-image technique and anti-mode method.

**Results:**

ARTI was found to decline from 1.9% (confidence interval: 1.7–2.1) at round I to 1.1% (confidence interval: 0.8–1.3) at round II at the rate of 8% per year during the intervening period.

**Conclusion:**

A significant reduction in the risk of tuberculous infection among children was observed between two rounds of surveys carried out at an interval of about 9 years.

## Introduction

The magnitude of Tuberculosis (TB) as a major health problem in India was revealed during a nationwide survey carried out during 1955–58 by the Indian Council of Medical research (ICMR) when the prevalence of bacteriologically positive pulmonary TB was found to be 400 per 100000 population [1]. The National TB program (NTP) implemented from the year 1962 failed to make any impact on the disease burden as revealed by serial surveys in defined geographical areas to find out the trends in prevalence of PTB [2]. Consequently, the Revised National TB Control Program (RNTCP) adopting DOTS (internationally recommended strategy to control TB) was launched in 1997 and gradually expanded to the entire country by 2006 [3].

To find out the recent trends in epidemiological situation of TB, two rounds of tuberculin surveys were carried out in four specifically defined geographical zones- north, south, east and west, with the objectives to estimate trends in annual risk of tuberculous infection (ARTI) among children in each of the zones. Each zone consisted of contiguous areas comprising one fourth of the country's population. While the ARTI rates among children without BCG (Bacille Calmatte Guerin) scar estimated at first rounds of surveys have been presented earlier [4]–[5], the estimates among children including those with BCG scar and the trend in ARTI between the two rounds of surveys in respect of North Zone are presented hereunder.

## Materials and Methods

Two rounds of surveys in northern zone were carried out during 2000–01 and 2009–10 respectively. Round I was planned to estimate ARTI among children without BCG scar [5]. However, children with BCG scar that were encountered during the process of registration were also tuberculin tested. Analysis of data revealed that estimate of ARTI among children without BCG scar was similar to the estimate among all children that included those with BCG scar [6]. Therefore, Round II was planned to estimate ARTI in all children. Both the Rounds were house based and were carried out among children 1–9 years of age.

Sample size for both rounds was calculated to estimate the prevalence of tuberculous infection with 10% relative precision at 5% level of significance, considering the design effect at 2. At Round I, sample size for children without BCG scar was estimated at 11045, arbitrarily considering the expected prevalence of infection at 10.0%. At Round II, sample size for all children was estimated at 13600, considering the prevalence of infection at 6.6% assuming a decline of 5% per year during the period intervening between the two rounds. Sample size thus estimated was equally allocated to 600 clusters at round I and 300 clusters at round II. The numbers of clusters were allocated in the ratio of population size to six districts selected by population proportional to size sampling technique (PPS). Within individual districts, numbers of clusters were further allocated to rural and urban areas in proportion to their population size; rural and urban clusters were selected using PPS.

Three visits were made to each cluster. During first visit, important community leaders were apprised of the purpose of the survey and field procedures. During the second visit, a rough sketch of the cluster was drawn and the lane to begin registration of children was selected using random number table. In the selected lane, registration commenced from one end of the lane and contiguous houses were visited in a clockwise direction. The registration team moved to the subsequently numbered lanes until the cluster size fixed at 85 during Round I and 45 during Round II was achieved. Tuberculin testing was performed on the same day at a temporarily setup centre within the cluster with written consent of the parent/guardian, after recording the BCG scar status. Each child was administered 0.1 ml of tuberculin dilution containing PPD RT23 intra-dermally on the mid -volar aspect of left forearm. While 1TU dose of PPD was used at round I; 2 TU was used at round II due to non-availability of 1 TU. Each test was recorded as “satisfactory” when a pale wheal with clear pits and well defined borders was raised. Otherwise it was recorded as “unsatisfactory”. Children with fever, skin rash or history of anti-TB treatment during last six months were excluded. Third visit was undertaken at about 72 hours after the test during which a trained reader identified the margins of indurations at the test site by palpation and recorded the maximum transverse diameter of induration in millimeters (mm). Double data entry was undertaken and the files were matched to find and correct any key punch errors.

At round II, in addition to children, 150 smear positive pulmonary TB (PTB) patients initiated on treatment during last one month were also tuberculin tested.

### Statistical Methods

Tuberculin reaction sizes in the study population were plotted as frequency distribution graphs for all children and also separately for children with and without BCG scar, in order to identify the mode of tuberculous reactions and the anti- mode that separates true tuberculous reactions from cross-reactions [7].

Prevalence of infection was estimated by Mirror image (MI)-method as well as anti-mode method [7]. In the former method, proportion of reactions larger than the mode of tuberculous reactions, is doubled and added to the proportion at the mode. In the latter method, all reactions larger than the anti-mode are considered tuberculous in nature.

In order to reduce the influence of digit preference in reading of tuberculin reaction sizes, 5-point moving averages were used for estimations.

ARTI was computed from the estimated prevalence of infection using the formula [8]:-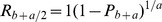
Where ‘b’ is median year of birth of children test-read, ‘a’ is mean age of children, P_b+a_ is prevalence of infection, R_b+a/2_ is ARTI at a mid-point in calendar time between median year of birth and the period of survey.

Average per year change (α) in ARTI between the two rounds was estimated for all children, using the formula: α = In[ ARI(t_1_)/ARI(t_0_)]/t_1_−t_0_), where In is the logorithmic value and t_0_ and t_1_ represent the years to which the estimated ARTI rates would correspond to, at rounds I and II respectively [9].

Estimates were also made by BCG scar status in order to find out the influence of BCG induced tuberculin sensitivity on ARTI estimates.

Analysis was also undertaken by Mixture Model using R Software and scripts available at www.tbrieder.org [10]. Since, the model did not generate a good fit, the results are not presented.

## Results

Numbers of children registered and satisfactorily test read at Round I and II are presented in table 1.

**Table 1:** Numbers of children investigated, Rounds I and II.

	No. registered	Excluded from testing	No. tested		No. read out of satisfactorily tested		
			Satisfactory	unsatisfactory	BCG−	BCG+	All@
Round I	55 433	3728 (6.7%)	51 380 (87.7%)	325	25 816	21 869	48 323 (94.1%)
Round II	15 175	1524 (10.0%)	13 309 (94.2%)	342	4286	7684	12 535 (94.2%)

*BCG−: children without BCG scar.*

*BCG+: children with BCG scar.*

@ *Includes children with doubtful scar.*

*( ): proportion excluded from testing out of registered children.*

*[ ]: proportion satisfactorily tested out of registered.*

*{ }: proportion read out of satisfactorily tested children.*

**Figure 1:**
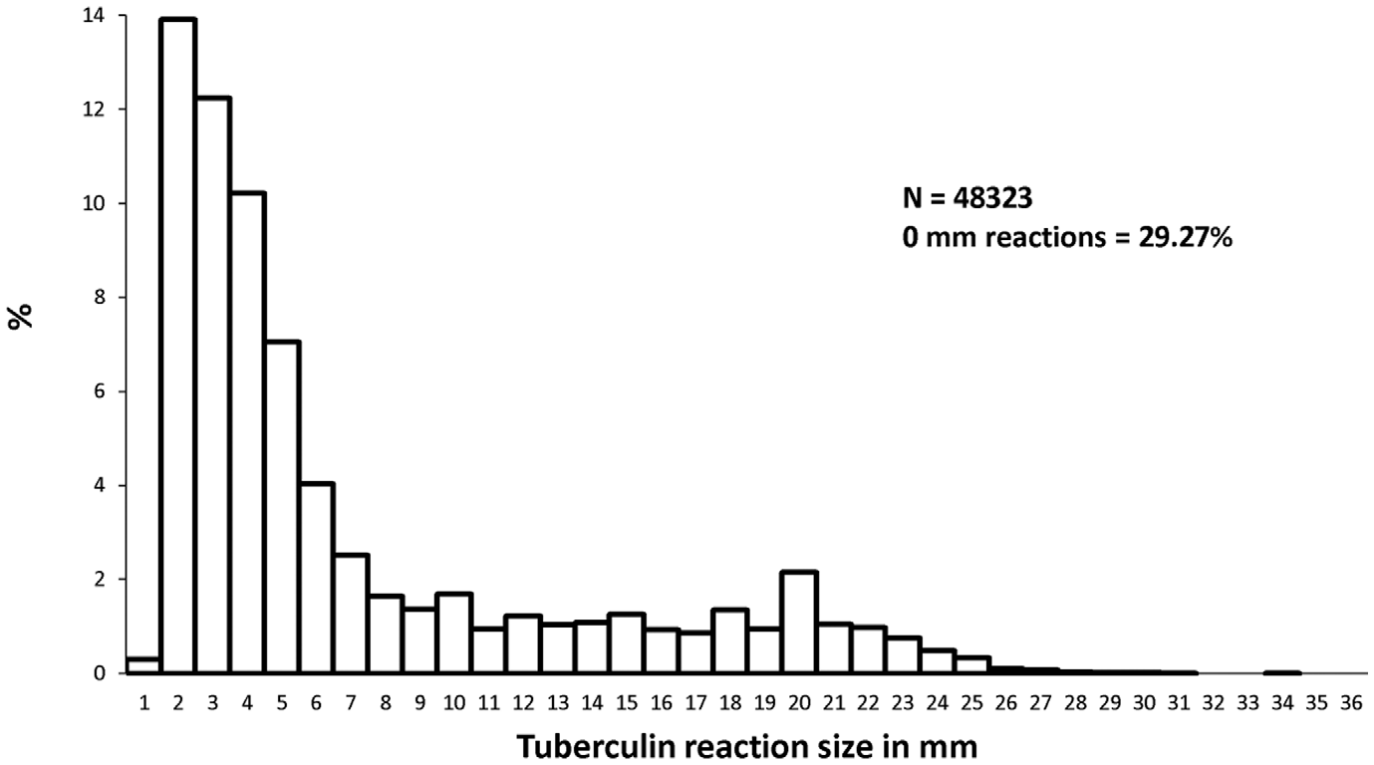
Frequency distribution of tuberculin reaction sizes. Survey I ; Stratum : Both ; BCG : Both; Age : 1–9 years.

**Figure 2:**
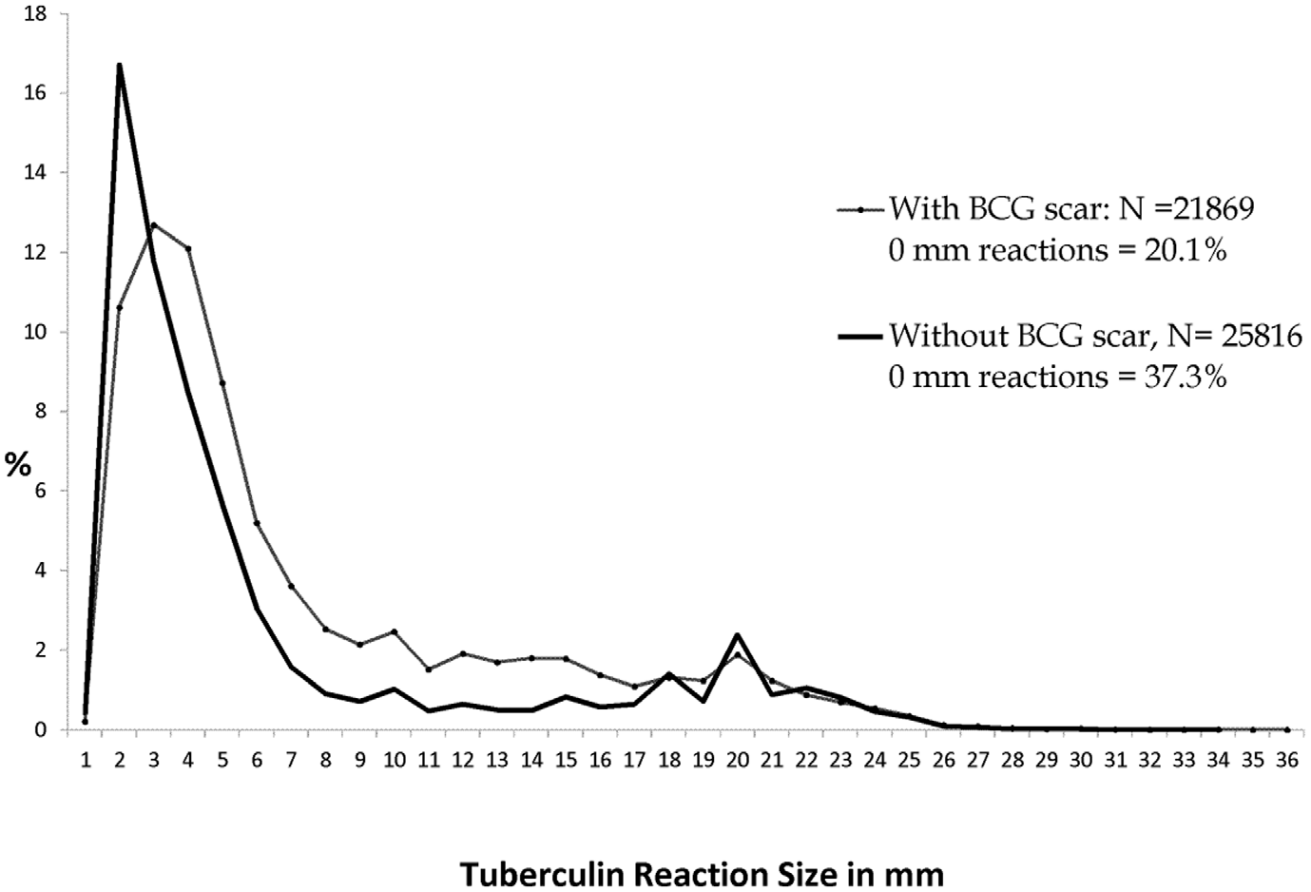
Frequency distributions of tuberculin reaction sizes by BCG scar status. Survey I ; Stratum: Both ; Age: 1–9 years.

At Round I, the mode of tuberculous reactions was discernible at 20 mm among all children as well as among children without BCG scar (Fig. 1, 2). An anti-mode at 14 mm was seen among children without BCG scar.

**Figure 3:**
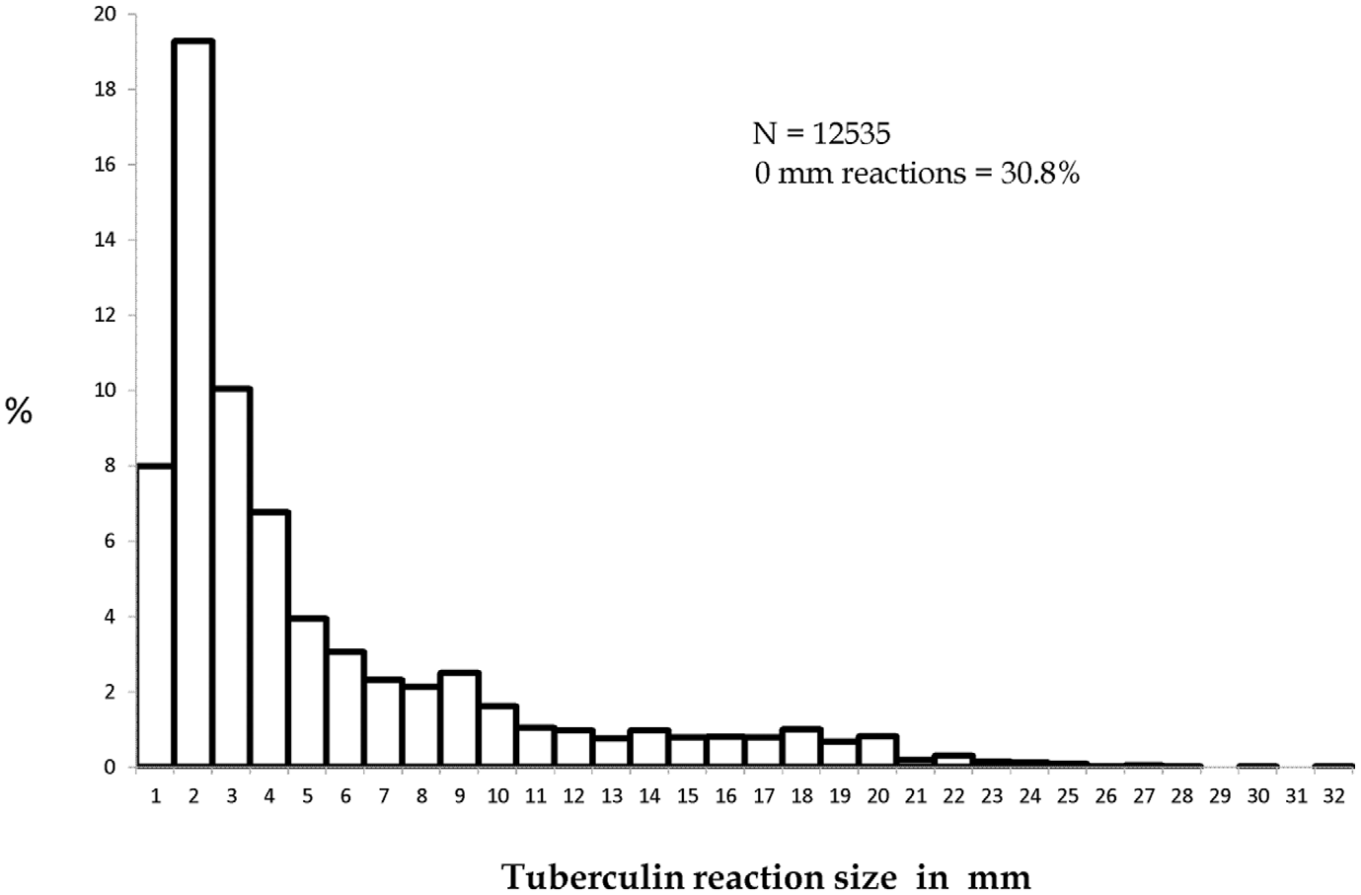
Frequency distribution of tuberculin reaction sizes. Survey II ; Stratum : Both ; BCG : Both ; Age : 1–9 years.

**Figure 4:**
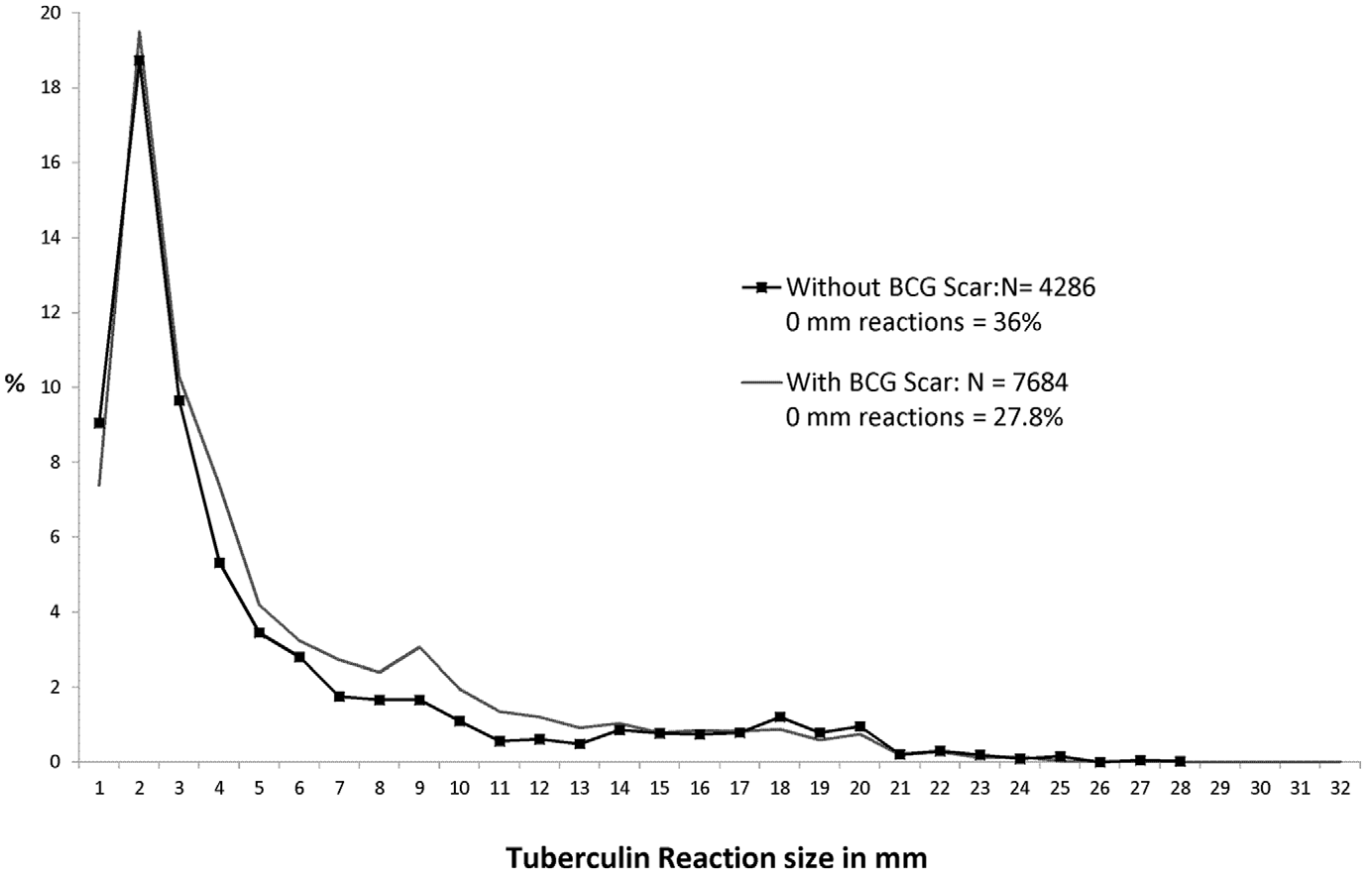
Frequency distribution tuberculin reaction sizes by BCG scar status. Survey II ; Stratum : Both ; Age : 1–9 years.

At Round II, the frequency distribution of reaction sizes did not reveal clear bi-modality among all children. Among children without BCG scar, mode of tuberculous reactions was discernible at 18 mm and an anti-mode at 13 mm (Fig. 3, 4).

Distributions of reaction sizes showed terminal digit preference especially at 20 mm during Round I; there was no suggestion of terminal digit preference at Round II.

Prevalence of infection among all children, using MI method was estimated at 10.1% (CI: 9.1–11.1) at Round I and 5.9% (CI: 4.7–7.0) at Round II. Mean age of all children was 5.62 years and 5.70 years at surveys I and II respectively. The computed ARTI rates were 1.9% (CI: 1.7–2.1) and 1.1% (CI: 0.8–1.3) respectively.

Prevalence of infection estimated by MI method was lower among children with BCG scar compared to without scar at both rounds; however the computed ARTI rates were similar between the two groups of children (Table 2). Among children without BCG scar, estimates by Anti-mode method were similar to those by MI method at both the rounds.

**Table 2:** Estimated prevalence of infection and ARTI by BCG scar status, Round I and II.

Method of Estimation		Round I		Round II	
		BCG −	BCG +	BCG −	BCG +
	No. test read	25816	21869	4286	7684
	Mean age (years)	5.76	5.45	6.05	5.51
Mirror image method	Prevalence (%)	10.9 (9.7–12.2)	9.5 (8.3–10.7)	6.7 (4.1–9.3)	5.4 (4.0–6.8)
	ARTI (%)	2.0 (1.8–2.1)	1.8 (1.6–2.0)	1.1 (0.7–1.6)	1.0 (0.7–1.3)
Anti-mode method	Prevalence (%)	11.4 (10.5–12.3)	-	7.6 (5.8–9.5)	-
	ARTI (%)	2.1 (1.9–2.2)	-	1.3 (1.0–1.6)	-

*BCG−: children without BCG scar.*

*BCG+: children with BCG scar.*

*( ): 95% confidence intervals.*

*N.B. Difference in Prevalence of infection between children without and with BCG scar was significant at both rounds value: survey I<0.001, survey II = 0.004.*

Estimates of ARTI by MI method revealed a decline of 8.0% per year in all children.

## Discussion

The ARTI rates among all children were estimated at 1.9% and 1.1% during round I and II respectively. These rates would apply to the years 1998 and 2007 respectively considering the mid-points of the surveys and mean age of the children surveyed. In 1998, RNTCP had just been introduced in the zone and its population coverage was merely 20%. Though the erstwhile National TB Programme was in vogue in the other areas, its performance was quite dismal in terms of case detection and case holding. Thus the observed decline in ARTI at 8% per year corresponded to the expansion phase of RNTCP. Similar declines of 6–8% have also been observed in two surveys carried out in Bangalore city and a rural area of Tamil Nadu state during the expansion phase of RNTCP and in other regions with expanding TB control programs [11]–[13]. Nevertheless, the observed ARTI of 1.1% during round II in the present study suggests on-going transmission of infection in the community. The ARTI rates in many of the developed countries have been observed to be below 0.1% [14].

Data analysis confirmed that BCG vaccinated children can be included in tuberculin surveys since the estimates ARTI rates were similar between children without and with BCG scar. Lower prevalence of infection among children with BCG scar compared to without scar was due to lower duration of exposure among the former since their mean age was found to be lower compared to children without BCG scar which may be attributed to disappearance of scar in a proportion of vaccinated children with age as observed in an earlier study [15]. There has been no change in BCG vaccination policy in the intervening years. Much credence may also not be heeded to different doses of PPD used at Round I and II, as both doses have been found to elicit similar tuberculin sensitivity patterns among the truly tuberculous infected populations [16].

In conclusion, the study results showed that the implementation of a DOTS-based programme for TB control was associated with a reduction in transmission of TB infection. However, the observed ARTI rate at the later survey is still a cause of concern and suggests continued intensification of TB control activities with increased vigour.
